# Hypothalamic ghrelin receptors, weight loss, and glycemia in an experimentally induced obesity model treated by sleeve gastrectomy

**DOI:** 10.1590/acb408025

**Published:** 2025-11-10

**Authors:** Claudia Gissi da Rocha Ferreira, André Richter Ribeiro, Christiane Madrid Finck, Ana Paula Percicote, Jorge Eduardo Fouto Matias

**Affiliations:** 1 Universidade Federal do Paraná – Departamento de Cirurgia – Pós-Graduação em Clínica Cirúrgica – Curitiba (PR), Brazil.; 2 Universidade Federal do Paraná – Departamento de Patologia – Curitiba (PR), Brazil.

**Keywords:** Obesity, Gastrectomy, Ghrelin, Receptors, Ghrelin

## Abstract

**Purpose:**

Body weight, blood glucose, and hypothalamic ghrelin receptors were monitored in an animal model of obesity after being treated with sleeve gastrectomy.

**Methods:**

Forty-two adult male Wistar rats were randomized into two groups: a non-obese group (standard chow); and an obese group, in which obesity was induced by feeding liquid enteral formula Ensure Plus. Each group was divided according to the surgery performed (sham operation or sleeve gastrectomy) and the time of sacrifice after surgery (14 or 28 days). Body weight and capillary blood glucose were monitored throughout the pre- and postoperative periods. Microscopic sections of the parietal cortex, thalamus, hypothalamus, and hippocampus were treated by immunohistochemical reaction with polyclonal anti-ghrelin receptor antibody. Positivity was determined by identifying labeled nuclei and cytoplasm in the brain cells.

**Results:**

Sleeve gastrectomy induced effective weight loss (*p* < 0.001) and reduction in the hypothalamic ghrelin receptor expression (*p* = 0.04). Weight loss was not directly influenced by the receptor expression. There was no significant impact on capillary glycemia.

**Conclusion:**

Sleeve gastrectomy alters GHSR1a receptors, decreasing their expression and body weight. However, weight loss is not directly related to the GHSR1a expression status.

## Introduction

Obesity is defined by the World Health Organization1 as an accumulation of body fat that leads to a body mass index (BMI) equal to or greater than 30 kg/m^2^. BMI values above 25 are directly related to increased mortality^1^. Populations around the world are turning progressively obese, which becomes a serious public health and economic issues^1^. Treatment obesity based on diet, physical activity, and other lifestyle changes has low success rates, resulting in 3 to 5% body weight loss^2^^–^ 4. Recently, the use of medications that simulate the action of some intestinal hormones such as glucagon-like peptide (GLP1) and gastrointestinal peptide (GIP) has obtained promising preliminary results in the pharmacological approach to the obesity^5^.

However, the gold standard treatment for obesity remains bariatric surgical procedures, the main techniques being Roux-en-Y gastric bypass and sleeve gastrectomy. Among them, sleeve gastrectomy has recently become the most worldwide used technique^6^. Although very distinct, these techniques produce similar clinical results concerning weight loss and early improvement of type II diabetes *mellitus* 7. These results, not yet completely understood, may be related to changes in the homeostatic control centers of the hypothalamus. In these nuclei, there are several groups of neurons responsive to different hormones, including those from the gastrointestinal tract, such as ghrelin.

Ghrelin is the only hormone known as being directly involved in the process that triggers the sensation of hunger^5^. In addition, ghrelin can act on long-term metabolic control through mechanisms that include modulation of food intake, energy expenditure, nutrient partitioning, adipocyte metabolism, and locomotor activity^8^^,^ 9. Ghrelin acts via binding to the growth hormone secretagogue receptor 1a (GHSR1a), distributed throughout several organs and particularly in the hypothalamic nuclei in the central nervous system^9^.

The present study aimed to investigate the possible relationships between weight loss, capillary glycemia, and hypothalamic ghrelin receptors in the setting of experimentally induced obesity treated by vertical gastrectomy.

## Methods

The study was submitted to the Universidade Federal do Paraná Animal Research Ethics Committee and approved under number 894, process no. 23075-079636.

Adult male Wistar rats weighing 250 to 300 g, aged between 12 and 16 weeks old, were housed in individual metabolic cages in a controlled environment of temperature (22°C) and relative humidity (45%) and under a 12-hour day-night cycle, with standard oral chow (Nuvital, Nuvilab) for 15 days. After the acclimatization period, under the effect of inhalation anesthesia with 4% isofluorane (Isoforine, Cristália), animals were weighed, had their capillary blood glucose measured (Accu-Chek Roche) in a drop of blood from the tail, and were randomized to induction or not of obesity by a hypercaloric diet.

### Obesity induction

Obesity was induced by oral feeding of a hypercaloric enteral nutrition formula (Ensure Plus, Abbott) with 54% carbohydrates, 17% proteins, 30% fats, and a caloric density of 1.5 kcal/mL. All animals were weighed and had their capillary blood glucose levels measured every four weeks. They were considered obese when their body weight exceeded the average weight of animals on a standard diet by at least 25%. All obese and non-obese animals were randomized to undergo sleeve gastrectomy (sleeve procedure) or the control procedure (sham procedure) in a 2:1 ratio, respectively.

### Operative procedures

After fasting for 24 hours, with only access to water, the animals underwent inhalation anesthesia induced in a closed chamber with vaporized isofluorane at a concentration of 4%. Anesthesia was maintained under spontaneous ventilation, using a mask with inhalation of 2% isofluorane and O2 at a flow rate of 1 to 2 L per minute. After trichotomy and skin antisepsis, a median longitudinal laparotomy of 4–5 cm in length was performed to access the peritoneal cavity.

In animals undergoing sleeve gastrectomy, the stomach was released and exteriorized. A 6F polyethylene catheter was introduced orally into the duodenum. The stomach was clamped next to the installed tube, excluding the greater gastric curvature, which was resected in approximately 80% of its length, preserving the esophagogastric junction and pylorus. Then, the posterior and anterior gastric walls were sutured in two continuous layers of polyglactin 910, 6-0 (Vicryl, Ethicom), the intragastric tube was removed, the stomach was returned to the cavity, and the incision was closed.

In the control group, the stomach was released, pulled, and exteriorized, and the 6F polyethylene catheter was introduced orally into the gastric cavity and directed to the duodenum. The sham procedure consisted of manipulation of the intestinal loops and inspection of the contents of the peritoneal cavity. Then, the probe was removed, the stomach returned to the abdominal cavity, and the procedure was completed by closing the abdominal wall in an identical manner as performed in animals undergoing sleeve gastrectomy.

At the end of each procedure, 0.9% saline solution (20 mL/kg of body weight) and dipyrone (160 mg/kg) were administered subcutaneously.

All animals that underwent surgery were kept fasting for 24 hours with access to water, and on the second postoperative day, a liquid diet (Ensure Plus, Abbott) was resumed for 24 hours. On the third day, all animals were reintroduced to a solid diet with the standard chow already mentioned. All animals were kept in individual metabolic cages and were observed and monitored for body weight and capillary blood glucose until sacrifice, 14 or 28 days after the surgical procedure.

During the sacrifice, under the same anesthesia protocol already mentioned, the abdominal incision was reopened, and access to the apex of the left ventricle was performed through a thoracotomy for circulatory perfusion, and the right atrium was incised to allow blood and perfusion fluid to drain. Using a peristaltic pump, 0.9% saline solution was infused for 10 minutes, followed by an infusion of 4% buffered formalin for another 10 minutes.

After the perfusion was completed, the skull was exposed and incised through posterior midline access along the superficial layers of the neck to the nose. After carefully incising and separating the lateral portion of the vault, the brain was delicately mobilized and removed in its entirety, weighed, and stored in a 10% formalin solution for later histological study.

### Immunohistochemistry

Serial coronal sections of 4 µm thickness were performed, focusing on sections of the parietal cortex, thalamus, hypothalamus, and hippocampus. All slices obtained were subjected to histological processing for analysis according to the method described by Rao et al.^10^.

After slides deparaffinization and dehydration/rehydration, the endogenous peroxidase was blocked by immersing them in distilled water and 5% hydrogen peroxide for 15 minutes. For antigen retrieval, the slides were immersed in ImmunoRetriver pH 9 in a water bath (96 to 99°C) for 30 minutes. Incubation was performed with the primary rabbit polyclonal antibody anti-ghrelin receptor (dilution 1:100/Abcam) overnight at a temperature between 2 and 8°C, in a wet chamber. The secondary antibody (Mouse/Rabbit PolyDetector DAB HRP Brown Detection System-Bio SB) was incubated for 40 minutes at room temperature. The DAB + substrate complex was used to develop the slides, and counterstaining was performed with Harris hematoxylin.

The selection of areas (hypothalamic nuclei) was carefully indicated by microscopic anatomy and guided by specific neuroanatomy images of the animal used^11^. Negative and positive controls were performed in parallel with all reactions^12^.

The positivity assessment was performed by identifying positive marking in the cytoplasm/nucleus of brain cells and locating the anatomical area according to the histology of the animals.

### Statistical analysis

The statistical analysis was performed based on a 3^2 factorial experimental design, with the following factors: obesity (obese or non-obese), surgery (sleeve gastrectomy or control), and time (14 or 28 days).

For the weight loss and blood glucose reduction, analysis of variance (3-way analysis of variance) was used, in which interactions between factors remained only when significant at 5% level. For the analysis of the GHSR1a receptor, logistic regression was used, with the factors obesity, type of surgery, and time after surgery, as previously described. Interactions between these three factors as covariates were inserted only if they were significant at the 5% level. From the adjusted models, contrasts between groups in each scenario were calculated, with *p*-values adjusted for multiple comparisons using the Bonferroni method. Differences between GHSR1a-negative and positive animals were compared using Student’s *t*-test.

Mediation analyses were performed to verify the direct effect of surgery, the effect mediated by the action of the GHSR1a receptor on weight loss, and the decreased blood glucose levels in the animals.

Statistical analysis used R software, version 4.2.3, and p-values were considered significant when equal to or less than 0.05.

## Results

### Induction of obesity

All animals using the hypercaloric diet achieved the criterion of gaining at least 25% more weight than the average weight of animals of the same age fed a standard diet. This required a period of 12 to 14 weeks on a hypercaloric liquid diet.

### Body weight

The animals’ weight was assessed at the time of surgery and at sacrifice, 14 or 28 days postoperatively (Table 1).

**Table 1:** Animals’ body weight during the experimental period.

Groups/subgroups	Pre-operative weight (g) (X ± SD)			Weight at sacrifice (g) (X ± SD)	
	14 days	28 days		14 days	28 days
**Obeses**					
Sleeve gastrectomy (n = 16)	460.0 ± 22.3	446.3 ± 79.7		388.1 ± 41.6	444.1 ± 32.8
Control (n = 8)	404.2 ± 10.0	427.5 ± 19.8		382.0 ± 16.1	434.2 ± 27.2
**Not obeses**					
Sleeve gastrectomy (n = 12)	359.0 ± 22.5	379.8 ± 8.8		331.5 ± 20.5	401.3 ± 15.0
Control (n = 6)	486.0 ± 57.1	446.3 ± 79.7		462.3 ± 48.4	470.0 ± 96.6

X ± SD: mean ± standard deviation. Source: Elaborated by the authors.

Sleeve gastrectomy was associated with greater weight loss, which was more pronounced at 14 days (Table 1).

Table 2 shows the statistical comparison of the weight loss by analysis of variance (3-way ANOVA).

**Table 2:** Analysis of variance (3-way ANOVA) for weight loss during the experiment.

Features	Beta	95%CI	*p* -value
Time (28 days)	-30.35	-58.81 - (-1.89)	0.037
Obese (yes)	-0.16	-29.85 - 29.54	0.991
Surgery (sleeve)	48.61	22.76 - 74.46	< 0.001
Time (28 days) × obese (yes)	-15.8	-44.88 - 14.51	0.306
Time (28 days) × surgery (sleeve)	-4.35	-35.52 - 26.82	0.779
Obese (yes) × surgery (sleeve)	-43.43	-74.93 - (-11.93)	0.008

95%CI: confidence interval of 95%. Source: Elaborated by the authors.

The factors time and surgery were preliminarily associated with weight loss in a significant way. When the factors were analyzed in double combinations, only the association between obese and surgery was able to show statistical significance concerning effective weight loss (*p* = 0.008).

### Capillary blood glucose

Table 3 shows the capillary blood glucose values measured on the day of surgery and on the days of sacrifice at 14 and 28 days postoperatively.

**Table 3:** Blood glucose in the study groups over time.

Groups/subgroups	Pre-operative blood glucose (mg/dL) (X ± SD)			Blood glucose at sacrifice (mg/dL) (X ± SD)	
	14 days	28 days		14 days	28 days
**Obeses**					
Sleeve gastrectomy (n = 16)	112.2 ± 10.0	96.0 ± 23.5		98.9 ± 15.8	99.1 ± 13.6
Control (n = 8)	99.2 ± 12.8	104.8 ± 6.7		90.0 ± 12.2	99.2 ± 8.5
**Not obeses**					
Sleeve gastrectomy (n = 12)	92.4 ± 24.7	102.8 ± 12.6		91.8 ± 12.7	107.5 ± 9.8
Control (n = 6)	119.7 ± 7.8	88.7 ± 14.2		103.3 ± 17.6	97.7 ± 16.3

X ± SD: mean ± standard deviation. Source: Elaborated by the authors.

The statistical comparative analysis for the decrease in blood glucose during the experiment is shown in Table 4.

**Table 4:** Analysis of variance (3-Way ANOVA) for capillary blood glucose variation over experiment time.

Features	Beta	95%CI	*p* -value
Time (28 days)	-12.14	-24.61 - 0.32	0.056
Obese (yes)	-5.99	-18.58 - 6.61	0.342
Surgery (Sleeve)	-3.71	-16.94 - 9.51	0.573

95%CI: confidence interval of 95%. Source: Elaborated by the authors.

There was no significant change in blood glucose levels in any of the comparisons or time points studied.

### Hypothalamic ghrelin receptors

**Figure 1:**
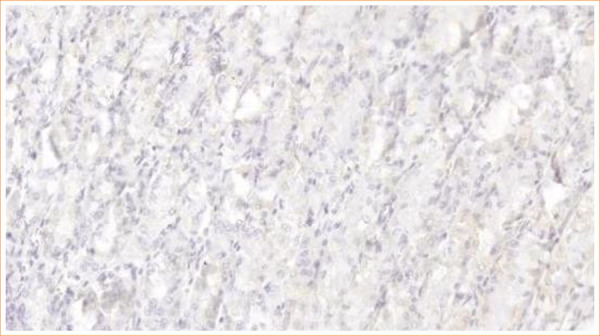
Immunohistochemistry for ghrelin receptors in the hypothalamus: negative result.

Source: Elaborated by the authors.

**Figure 2:**
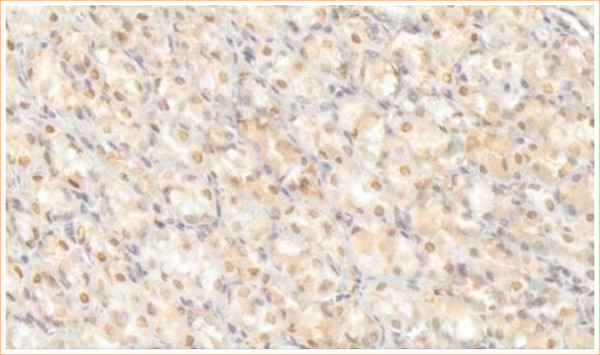
Immunohistochemistry for ghrelin receptors in the hypothalamus: positive result.

Source: Elaborated by the authors.

Figures 1 and 2 illustrate, respectively, the negative and positive results from the immunohistochemical expression study of the GHSR1a receptor in the hypothalamic nuclei of the animals in the study.

The expression of the GHSR1a receptor in obese animals submitted to sleeve gastrectomy was 37.5% for both periods of 14 and 28 days after the procedure. The obese animals that underwent a sham procedure presented GHSR1a receptors in 100% of the animals at 14 days and 75% at 28 days (Table 5).

**Table 5:** Hypothalamic growth hormone secretagogue receptor 1a (GHSR1a) expression in the study groups over time.

Groups/subgroups	Percentage of expression of the hypothalamic ghrelin receptor (GHSR1a)	
	14 days (%)	28 days (%)
**Obeses**		
Sleeve gastrectomy (n = 16)	37.5	37.5
Control (n = 8)	100	75
**Not obeses**		
Sleeve gastrectomy (n = 12)	16.7	33.3
Control (n = 6)	66.7	0

Source: Elaborated by the authors.

Logistic regression analysis demonstrated that sleeve gastrectomy was associated with a lower chance of positive GHSR1a, when adjusted for time of sacrifice and obesity (*odds ratio*—OR = 0.23; 95% confidence interval—95%CI 0.05–0.92; *p* = 0.046) (Table 6).

**Table 6:** Logistic regression analysis for growth hormone secretagogue receptor 1a (GHSR1a) expression.

Features	Odds ratio	95%CI	*p* -value
Time (28 days)	0.63	0.16–2.40	0.497
Obese (yes)	3.57	0.92–16.4	0.078
Surgery (sleeve)	0.23	0.05–0.92	0.046

95%CI: confidence interval of 95%. Source: Elaborated by the authors.

Through the mediation analysis performed, it was observed that the effect of sleeve procedure on weight loss was mostly direct, with no significant effect mediated by the GHSR1a status, as demonstrated in Table 7.

**Table 7:** Association between sleeve gastrectomy and weight loss, mediated by growth hormone secretagogue receptor 1a (GHSR1a) expression.

Effects	Estimate	95%CI	*p* -value
Direct effect	25.03	(2.86–45.66)	0.026
Indirect effect	3.52	(-4.96–15.60)	0.462
Full effect	28.55	(9.81–46.84)	< 0.001
Proportion of total mediated effect	0.123	(-0.212–0.760)	0.462

95%CI: confidence interval of 95%. Source: Elaborated by the authors.

## Discussion

Our choice for the experimental obesity model induced obesity through a hypercaloric diet, and the sleeve procedure performed was effective in inducing weight loss, as already stated before^13^^,^ 14. We also observed the attenuation of weight loss over time among both obese and non-obese animals, again in line with other studies^14^^,^ 15. However, there was no significant change in fasting blood glucose in animals that developed obesity and were submitted to sleeve gastrectomy. Sadie-Van Gijsen and Kotzé-Hörstmann^16^ analyzed 102 studies and noted the absence of changes in fasting blood glucose in 33% of them, which represents a significant divergence concerning the expected result of glucose metabolism in this research scenario.

Obesity induces changes of the hypothalamus nuclei in the central nervous system (CNS), as well as in the plasma levels of hormones and peptides involved in the control of metabolism and energy expenditure^17^^–^ 19.

In a recent detailed review, peripheral ghrelin blood levels were reduced after sleeve gastrectomy, in a period of up to 12 months, and remained unchanged or even elevated after Roux-en-Y gastric bypass, for up to 24 months postoperatively^20^. It is assumed that this difference corresponds to the removal of a large part of the stomach when gastrectomy is performed, since this is the main organ producing ghrelin. In the case of gastric bypass, the stomach is only excluded, maintaining the main source of production of this hormone. However, in a review of 45 studies with one to 24 months of follow-up after RYGB^20^, the plasma levels of ghrelin were unchanged or even increased, raising concerns about the role of peripheral hormones on the consequences of bariatric procedures. Likewise, the intricate functioning of GHSR1a, which includes ligand-independent activity and the ability to dimerize with several other receptors, represents a major challenge and obstacle to the search for a comprehensive understanding of its action mechanism 21.

Sleeve gastrectomy can induce changes in the central expression of brain GHSR1a, and this technique is related to the central expression downregulation of this receptor^22^. As a result of the current research, the decrease in receptor expression is significantly related to the time intervals. Through mediation analysis, we observed that this reduction in the receptor is not directly correlated to weight loss, the effect being from the surgical technique directly on weight loss occurring mainly early (14 days) and independently of the ghrelin receptor status. Up to now, no other study has found such a dissociated relationship between weight loss and GHSR1a expression after sleeve gastrectomy.

Although many publications addressing the physiology and pathophysiology of metabolic control exerted by CNS, the interactions between brain changes triggered by bariatric surgeries and changes in brain receptors for metabolic and behavioral control are still unclear^23^. The proposed effects range from benefits such as reduction of the inflammatory process induced by obesity in the CNS, recovery of cognitive function, and improvement in food choices to adverse consequences such as the development of mental disorders and alcoholism^24^^,^ 25.

Therefore, understanding the behavior of metabolic brain receptors may provide a glimpse into the development of molecules linked not only to the treatment of obesity, but also to diseases related to drug abuse^26^.

No significant change was detected in animals’ glycemic levels at any time during the experiment, which impaired comparisons with glycemic control resulting from bariatric techniques and the expression of GHSR1a in the hypothalamus. The lack of animals submitted to the Roux-en-Y gastroplasty technique for comparisons regarding the change in GHSR1a receptors and other parameters limited the possibility of comparing these two techniques regarding the role of ghrelin receptor in the CNS.

The role of GHSR1a receptor and its weight loss modulation and glycemic control remains uncertain, as does the possibility of its use in the treatment of diabetes, obesity, cachexia, and anorexia^27^. Due to the high intrinsic activity of this receptor, the level of expression of GHSR1a may be more relevant than its relationship with plasma ghrelin levels or its binding to this protein. The contradictory results obtained from research trying to understand plasma ghrelin’s relationship to appetite, weight loss, glycemia, and the type of surgery demand the need for in-depth knowledge of the receptor’s activity. Since ghrelin is the main hormone that binds to GHSR1a receptor and has the same close relationship with the maintenance of adequate glycemia levels, especially in stressful situations, it is reasonable to look for a relationship between the linked ghrelin/GHSR1a and the brain insulin receptors^28^^,^ 29.

Studies that correlate sleeve gastrectomy and Roux-en-Y gastric bypass with brain receptors not only for ghrelin (GHSR1a), but also with brain insulin receptors, may provide data to elucidate the relationship between metabolic/bariatric surgical techniques and the improvement of diabetes *mellitus* in operated obese patients.

There is still demand for research protocols that aim to gather new knowledge capable of supporting more powerful hypotheses about the role of the various biochemical and molecular agents responsible for the effects and regulation of organic responses to metabolic/bariatric surgical procedures.

## Conclusion

Sleeve gastrectomy reduces the expression of hypothalamic receptors for ghrelin (GHSR1a). This effect is influenced by the time elapsed after surgical treatment of obesity.

The weight loss that occurs after sleeve gastrectomy is not mediated by the reduction in GHSR1a expression in the hypothalamus, but rather by the direct effect of the bariatric procedure performed.

The variation in blood glucose levels throughout the study and between groups was not influenced by the GHSR1a expression status.

## Data Availability

All data can be obtained from the corresponding author.
